# Autosomal dominant tubulointerstitial kidney disease-UMOD is the most frequent non polycystic genetic kidney disease

**DOI:** 10.1186/s12882-018-1107-y

**Published:** 2018-10-30

**Authors:** Christine Gast, Anthony Marinaki, Monica Arenas-Hernandez, Sara Campbell, Eleanor G. Seaby, Reuben J. Pengelly, Daniel P. Gale, Thomas M. Connor, David J. Bunyan, Kateřina Hodaňová, Martina Živná, Stanislav Kmoch, Sarah Ennis, G. Venkat-Raman

**Affiliations:** 1 Wessex Kidney Centre, Queen Alexandra Hospital, Portsmouth Hospitals NHS Trust, Southwick Hill Road, Cosham, Portsmouth, PO6 3LY UK; 2 Human Genetics and Genomic Medicine, Faculty of Medicine, University of Southampton, Southampton, UK; 3 Purine Research Laboratory, Guys and St Thomas’ NHS Foundation Trust, London, UK; 4 UCL Centre for Nephrology, Royal Free Hospital, London, UK; 5 Oxford Kidney Unit, Churchill Hospital, Oxford, UK; 6 Wessex Regional Genetics Laboratory, Salisbury NHS Foundation Trust, Salisbury, UK; 7 Research Unit for Rare Diseases, Department of Pediatrics and Adolescent Medicine, First Faculty of Medicine, Charles University Prague, Prague, Czech Republic

**Keywords:** Genetic kidney disease, Autosomal dominant tubulointerstitial kidney disease, UMOD, Prevalence

## Abstract

**Background:**

Autosomal dominant tubulointerstitial kidney disease (ADTKD) caused by mutations in the *UMOD* gene (ADTKD-UMOD) is considered rare and often remains unrecognised. We aimed to establish the prevalence of genetic kidney diseases, ADTKD and ADTKD-UMOD in adult chronic kidney disease (CKD) patients, and to investigate characteristic features.

**Methods:**

We sent questionnaires on family history to all patients with CKD stages 3–5 in our tertiary renal centre to identify patients with inherited renal disease. Details on clinical and family history were obtained from patient interviews and clinical records. Sanger sequencing of the *UMOD* gene was performed from blood or saliva samples.

**Results:**

2027 of 3770 sent questionnaires were returned. 459 patients reported a family history, which was consistent with inherited kidney disease in 217 patients. 182 non-responders with inherited kidney diseases were identified through a database search. Of these 399 individuals, 252 had autosomal dominant polycystic kidney disease (ADPKD), 28 had ADTKD, 25 had Alports, and 44 were unknown, resulting in 11% of CKD 3–5 patients and 19% of end-stage renal disease patients with genetic kidney diseases. Of the unknown, 40 were genotyped, of whom 31 had findings consistent with ADTKD. 30% of unknowns and 39% of unknowns with ADTKD had *UMOD* mutations. Altogether, 35 individuals from 18 families were found to have ten distinct *UMOD* mutations (three novel), making up 1% of patients with CKD 3–5, 2% of patients with end-stage renal disease, 9% of inherited kidney diseases and 56% with ADTKD. ADTKD-UMOD was the most common genetic kidney disease after ADPKD with a population prevalence of 9 per million. Less proteinuria and haematuria, but not hyperuricaemia or gout were predictive of ADTKD-UMOD. The main limitations of the study are the single-centre design and a predominantly Caucasian population.

**Conclusions:**

The prevalence of genetic kidney diseases and ADTKD-UMOD is significantly higher than previously described. Clinical features poorly predicted ADTKD-UMOD, highlighting the need for genetic testing guided by family history alone.

**Electronic supplementary material:**

The online version of this article (10.1186/s12882-018-1107-y) contains supplementary material, which is available to authorized users.

## Background

Autosomal dominant tubulointerstitial kidney disease (ADTKD) is a rare genetic kidney disease. ADTKD caused by mutations in the *UMOD* gene (ADTKD-UMOD) is the most common form of ADTKD [1, 2]. Other gene mutations causing ADTKD include mucin 1 (*MUC1*), hepatocyte nuclear factor 1 beta (*HNF1b*), renin (*REN*), and the alpha subunit of the endoplasmic reticular membrane translocon (*SEC61A1*) [3–7]. Previously known as familial juvenile hyperuricaemic nephropathy (FJHN) and uromodulin associated kidney disease (UAKD), ADTKD-UMOD is characterised by early onset hyperuricaemia and gout affecting both sexes, and the development of insidious renal failure with tubulointerstitial disease [8]. These disorders characteristically do not feature haematuria or proteinuria. Patients usually develop end stage renal disease (ESRD) between the third and sixth decade of life. However, clinical features are variable and hyperuricaemia and gout may be absent [9]. Some patients are found to have medullary renal cysts [10]. It has been shown that pathogenic *UMOD* mutations cause protein misfolding, retention in the endoplasmic reticulum (ER) and mistargeting of uromodulin in the thick ascending limb of Henle, resulting in tubulointerstitial damage through ER stress and reduced urinary uromodulin excretion [11–13]. A recent knock-in mouse model harbouring a human mutation has given insight into the pathophysiology of ADTKD-UMOD [14].

Inherited interstitial kidney diseases are underrecognised and underreported due to their lack of distinctive clinical or diagnostic histological features, lack of physician awareness and incomplete acquisition of family histories [15]. Registry data reliant on accurate diagnostic coding is known to be incomplete [16], and there is a paucity of information on the prevalence of genetic kidney diseases. Published studies suggest a prevalence of polycystic kidney disease of 5–11% and of other familial nephropathies of 4–6% amongst ESRD patients [17–21]. Registry figures for the latter are even lower between 2 and 3% [22, 23]. The UK’s National Rare Disease Registry (RaDaR) lists 115 patients with ADTKD for a population of 65 million, resulting in a prevalence of 1.8 per million [24]. The published population prevalence of ADTKD-UMOD from a single Austrian study was 1.7 per million [25].

Preliminary data from our tertiary renal and transplant centre with a catchment population of 2 million had suggested that this was a gross underestimate, with a much higher in centre prevalence of ADTKD-UMOD [26]. Therefore we aimed to establish the prevalence of ADTKD-UMOD and genetic kidney diseases as a whole, and to investigate clinical and biochemical characteristics that may aid the recognition of ADTKD-UMOD.

## Methods

### Patient ascertainment

Questionnaires were sent to all patients in CKD stages 3–5 and all transplant recipients registered on the electronic database Proton. The one-page questionnaire asked patients to record any family members with kidney disease, their relation to the patient, their renal diagnosis (if known), the patient’s own diagnosis, and their willingness to be contacted about the study (Additional File 1). Genetic kidney diseases of interest to this study were defined as monogenic diseases rather than disorders of polygenic risk alleles predisposing to kidney disease. Positive responses were reviewed.

Non-responders with CKD stages 3–5 with a family history of renal disease were identified through a search of diagnostic codes, electronic patient letters and through their nephrologists. Patient letters were reviewed for the presence of a positive family history for all non-responders with missing diagnostic codes, diagnostic codes 0 (chronic renal failure, aetiology uncertain), 30 (interstitial nephritis due to other cause, or unspecified), 40 (cystic kidney disease, type unspecified), 43 (medullary cystic kidney disease including nephronophthisis), 49 (cystic kidney disease, other specified type), 50 (hereditary/familial nephropathy type unspecified), 59 (hereditary nephropathy, other specified type) and 92 (gout).

If a genetic kidney disease was likely as suggested by a relative with a compatible diagnosis, patients were invited to participate in the study.

Patients gave written informed consent before providing a blood or saliva sample. Clinical data and pedigrees were recorded from patient interviews and clinical records.

### Genetic investigations

Genomic DNA extraction from whole blood was performed by QIAamp DNA Blood Midi kit (Qiagen, Venlo, Netherlands) or the salting out method [27], and from saliva by Oragene kit (DNA Genotek, Ontario, Canada). Exons 3 to 5 of the *UMOD* gene were sequenced in an ABI 3130 XL Genetic Analyser. In three families, other *UMOD* exons had been sequenced beforehand by custom gene panel [6] or Sanger sequencing [13] at the Institute for Inherited Metabolic Disorders in Prague. Sequencing files were analysed by the software “Mutation Surveyor” (SoftGenetics, State College, PA, USA) using Genbank reference sequence NM_003361.3. Genetic variants were annotated with variant coding effects, predictive metrics of deleteriousness Polyphen-2 [28], and SIFT [29], and minor allele frequencies from the 1000 Genomes Project (1KG) [30], Exome Sequencing Project (ESP) [31] and Exome Aggregation Consortium (ExAC) [32] with ANNOVAR v2013Aug23 [33].

Exome sequencing of six samples from participants with a particularly strong family history was performed using the exome capture kits Agilent SureSelect v.5.0 (51 Mb) and Agilent Sure Select All Exon and sequenced on the HiSeq 2000 Sequencer or Illumina Genome Analyzer IIx. Reads were aligned to the reference genome (GRCh37) using Novoalign (Novocraft, 2010). Variants were called using GATK and annotated using Annovar.

Clinical confirmatory sequencing was performed using fresh blood samples.

### Features associated with ADTKD-UMOD

Clinical and biochemical features were compared between patients with pathogenic *UMOD* mutations and the remaining cohort. Statistical significance was determined by the χ^2^ test, Fisher’s Exact test, Mann-Whitney-U test, or Kruskall-Wallis test, as appropriate, using SPSS version 24 (IBM, Armonk, NY).

## Results

### Patient ascertainment

**Fig. 1:**
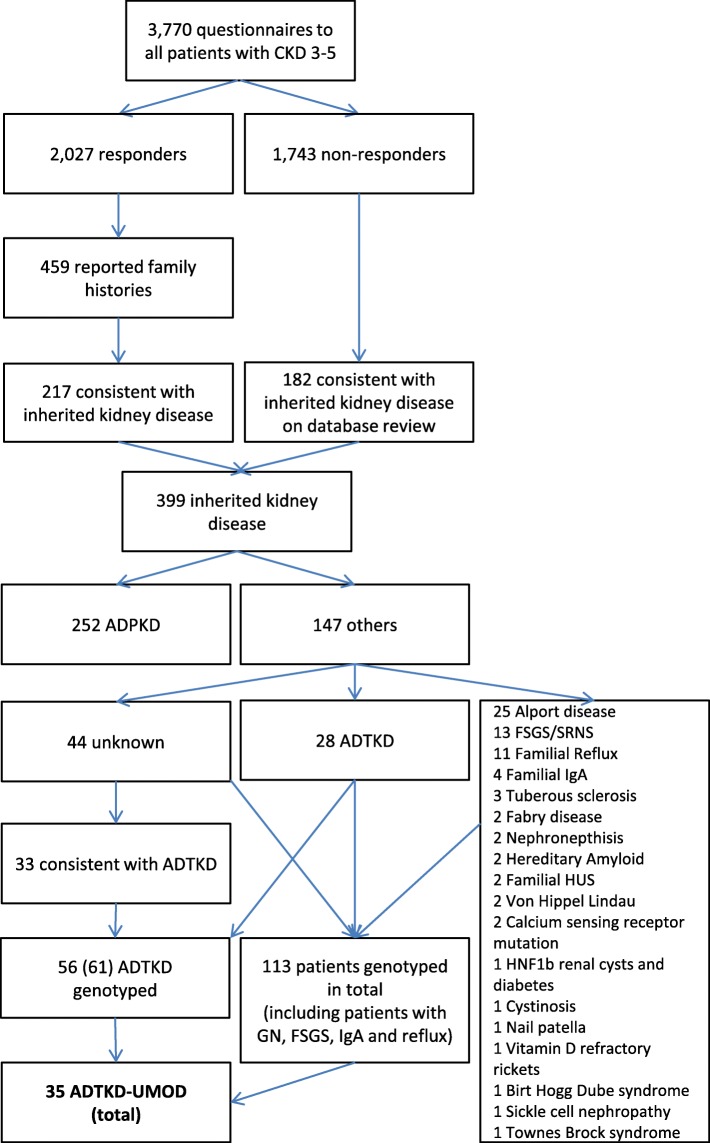
Diagnostic Pathway

3770 questionnaires on family history were sent to all patients (96% Caucasian) in CKD stages 3 to 5 and all transplant recipients registered on our electronic renal database. 2027 responses were received, corresponding to a response rate of 53.8%. 459 patients (22.6% of responders) reported a family history of kidney disease. Of these, in 217 patients (47%) an underlying genetic kidney disease was likely, in 184 patients (40%), the respective renal diagnoses for patients and relatives were apparently unrelated (e.g. diabetic nephropathy and renal cancer), and in 58 patients (13%) not enough information was available to allow an assessment (Fig. 1). The questionnaire study resulted in the identification of an additional 54 patients with genetic kidney diseases for whom either the diagnosis or coding were incomplete. Responders with an underlying genetic kidney disease were statistically younger (median age 59 versus 64 years with a reported family history and 68 years for all responders, *p* < 0.001, Kruskall-Wallis = 99.057), more likely to have ESRD (67% versus 38% for all responders, p < 0.001, χ^2^ = 79.827), and female (55% versus 41%, p < 0.001, χ^2^ = 79.827).

Amongst the non-responders, an additional 182 patients with genetic kidney diseases were identified through their nephrologists, a review of coded diagnoses and diagnoses extracted from clinic letters (Fig. 1). 38 of 61 patients (62%) with ADTKD had returned the questionnaire, and of these, 30 (79%) would have been identified by database screening.

### Prevalence of genetic kidney diseases

Of the total 399 patients with genetic kidney diseases, 252 (63%) had autosomal dominant polycystic kidney disease (ADPKD), and 147 (37%) had other genetic kidney diseases. For the latter cohort, the most common diagnoses were unknown familial nephropathies, followed by ADTKD, Alport disease, familial focal segmental glomerulosclerosis (FSGS) or steroid resistant nephrotic syndrome (SRNS), and familial reflux nephropathy.

The prevalence rate of genetic kidney diseases was 11% for all CKD 3+ patients and 19% for patients with ESRD, with very similar rates obtained for responders and all patients (Table 1), confirming the effective uncovering of patients with genetic kidney diseases amongst the non-responders. Genetic kidney diseases other than ADPKD had a prevalence rate of 4% amongst CKD patients and 8% amongst ESRD patients.

**Table 1:** Prevalence rates for genetic kidney diseases (GKD) in CKD cohort

	Responders	All Patients	Responders with ESRD	All patients with ESRD
All GKD	217/2027 = 10.7%	399/3770 = 10.6%	144/772 = 18.7%	269/1425 = 18.9%
ADPKD	140/2027 = 6.9%	252/3770 = 6.7%	88/772 = 11.4%	161/1425 = 11.3%
Other GKD (non-ADPKD)	77/2027 = 3.8%	147/3770 = 3.9%	56/772 = 7.3%	108/1425 = 7.6%
ADTKD	39/2027 = 1.9%	61/3770 = 1.6%	31/772 = 4.0%	44/1425 = 3.1%
ADTKD-UMOD	19/2027 = 0.9%	35/3770 = 0.9%	18/772 = 2.3%	27/1425 = 1.9%

### *UMOD* mutations

Sanger sequencing of exons 3–5 of the *UMOD* gene was performed on DNA samples from 113 participants with no established conflicting genetic diagnosis. Six participants from five families with a strong family history of renal disease underwent exome sequencing before Sanger sequencing. In three families this identified pathogenic *UMOD* mutations within exons 3–5 which were confirmed to segregate with disease by Sanger sequencing.

Ten distinct heterozygous gene mutations were found in 35 participants from 18 families, all Caucasian (Table 2). Two individuals from two families carried the non-disease causing variant p.(Thr62Pro). We achieved a new diagnosis of ADTKD-UMOD in 11 individuals from seven families and confirmed ADTKD-UMOD in 24 individuals from eleven families.

**Table 2:** *UMOD* mutation table

Study Number	Mutation (reference sequence NM_003361.3)	Protein Alteration	Family history of renal disease	Diagnosis of FJHN/ ADTKD-UMOD prior to study	Age at RRT	Hyperuricaemia	Gout
FN68 301^a^	c.202G > A	p.(Glu68Lys)	Yes	Yes	41	No	No
FN9 304^a^	c.263G > T	p.(Gly88Val)	Yes	Yes	44	Yes	No
FN2 301	c.272-274del	p.(Ser91del)	Yes	Yes	39	Yes	Yes
FN2 302	c.272-274del	p.(Ser91del)	Yes	Yes	45	Yes	No
FN2 303^a^	c.272-274del	p.(Ser91del)	Yes	Yes	58	Yes	No
FN3 301^a^	c.272-274del	p.(Ser91del)	Yes	No		Yes	No
FN3 305^a^	c.272-274del	p.(Ser91del)	Yes	No	64		No
FN3 409^a^	c.272-274del	p.(Ser91del)	Yes	No	38	No	No
FN26 301^a^	c.272-274del	p.(Ser91del)	Yes	No	56	Yes	No
FN45 304^a^	c.272-274del	p.(Ser91del)	Yes	Yes	45	Yes	No
FN45 404^a^	c.272-274del	p.(Ser91del)	Yes	Yes	51	No	Yes
FN45 405^a^	c.272-274del	p.(Ser91del)	Yes	Yes	37		No
FN65 201	c.272-274del	p.(Ser91del)	Yes	Yes	66		No
FN65 202	c.272-274del	p.(Ser91del)	Yes	Yes	59		No
FN65 203^a^	c.272-274del	p.(Ser91del)	Yes	Yes	59	Yes	No
FN65 301	c.272-274del	p.(Ser91del)	Yes	Yes		Yes	Yes
FN65 401	c.272-274del	p.(Ser91del)	Yes	Yes	47	Yes	Yes
FN65 402	c.272-274del	p.(Ser91del)	Yes	Yes		Yes	Yes
FN65 412^a^	c.272-274del	p.(Ser91del)	Yes	Yes	54	No	Yes
FN1 303^a^	*c.278_289delinsCCGCCTCCT*	p.(Val93_Gly97delinsAlaAlaSerCys)	Yes	No	62	Yes	No
FN24 305^a^	*c.278_289delinsCCGCCTCCT*	p.(Val93_Gly97delinsAlaAlaSerCys)	Yes	No	42	Yes	No
FN47 404^a^	*c.278_289delinsCCGCCTCCT*	p.(Val93_Gly97delinsAlaAlaSerCys)	Yes	Yes	49	Yes	No
FN77 301^a^	*c.278_289delinsCCGCCTCCT*	p.(Val93_Gly97delinsAlaAlaSerCys)	Yes	Yes	49	Yes	No
FN20 302	c.443G > A	p.(Cys148Tyr)	Yes	Yes	50	Yes	Yes
FN20 403	c.443G > A	p.(Cys148Tyr)	Yes	Yes		Yes	No
FN64 303^a^	c.614 T > C	p.(Phe205Ser)	Yes	No	42	Yes	Yes
FN23 302	c.629G > A	p.(Gly210Asp)	Yes	No	36	Yes	Yes
FN23 303	c.629G > A	p.(Gly210Asp)	Yes	No	46		No
FN27 304^a^	c.688 T > C	p.(Trp230Arg)	Yes	No	63	Yes	Yes
FN27 306^a^	c.688 T > C	p.(Trp230Arg)	Yes	No		Yes	Yes
FN28 302	c.688 T > C	p.(Trp230Arg)	Yes	Yes	57	Yes	Yes
FN28 401^a^	c.688 T > C	p.(Trp230Arg)	Yes	Yes		Yes	Yes
FN7 305	c.860G > A	p.(Cys287Tyr)	Yes	Yes	27	Yes	Yes
FN35 403^a^	c.917G > A	p.(Cys287Tyr)	Yes	Yes	57	Yes	No
FN35 501^a^	c.917G > A	p.(Cys287Tyr)	Yes	Yes	42	Yes	Yes

*RRT* renal replacement therapy, *FJHN* familial juvenile hyperuricaemic nephropathy

^a^clinically confirmed

28 patients had pre-existing diagnoses within the spectrum of ADTKD, 44 had unknown familial nephropathies, of whom 33 had clinical features consistent with ADTKD. Of the 44 unknown, 40 were genotyped. 30% (13/44) of the unknowns, 39% (13/33) of unknowns with ADTKD, and 57% (35/61) of all ATKD patients had *UMOD* mutations (Fig. 1). Altogether ADTKD-UMOD made up 1% (35/3770) of CKD 3+, 2% (29/1425) of ESRD, 9% (35/399) of inherited kidney diseases (24% without ADPKD), and 57% (35/61) of ADTKD. ADTKD made up 2% (61/3770) of CKD3+, 3% of ESRD (41/1425) and 15% (61/399) of inherited kidney diseases (42% without ADPKD).

Seven of the ten distinct gene mutations were published mutations and listed in the Wake Forest Inherited Kidney Disease Database for uromodulin associated kidney disease [34] (variant p.(Thr62Pro) was listed as clinically silent), and three of these were also present in the Human Gene Mutation Database (Table 3) [35].

**Table 3:** *UMOD* mutation characteristics

Mutation	Exon	Protein alteration	Wake Forest Registry	HGMD	Polyphen	SIFT	1KG	ESP	ExAC
c.184A > C^a^	3	p.(Thr62Pro) [34]	Yes^a^	No	0.662	0.030.03 0.	–	0.0006.006	0.0004
c.202G > A	3	p.(Glu68Lys) [34]	Yes	No	0.999	0	–	–	–
c.263G > T	3	p.(Gly88Val)	No	No	1	0	–	–	–
c.272-274del	3	p.(Ser91del) [34]	Yes	No			–	–	–
*c.278_289delinsCCGCCTCCT*	3	p.(Val93_Gly97delins AlaAlaSerCys) [42]	Yes	Yes			–	–	–
c.443G > A	3	p.(Cys148Tyr) [10]	Yes	Yes	1	0.31	–	–	–
c.614 T > C	3	p.(Phe205Ser)	No	No	1	0	–	–	–
c.629G > A	3	p.(Gly210Asp) [34]	Yes	No	1	0	–	–	–
c.688 T > C	3	p.(Trp230Arg) [43]	Yes	Yes	1	0	–	–	–
c.860G > A	3	p.(Cys287Tyr)	No	No	1	0	–	–	–
c.917G > A	4	p.(Cys306Tyr) [34]	Yes	No	1	0	–	–	–

Mutation = *UMOD* mutation, Wake Forest Registry = inclusion in the Wake Forest School Registry of Inherited Kidney Diseases, HGMD = inclusion in the Human Gene Mutation Database. Polyphen and SIFT = predictive scores of deleteriousness, 1 KG / ESP/ ExAC = occurrence in the large sequencing projects of populations with European ancestry 1000 Genomes (1KG) and Exome Sequencing Project (ESP) and in 60,000 healthy individuals from varying ethnicities in the Exome Aggregation Consortium (ExAC). ^a^clinically silent

The three novel mutations were classed as probably pathogenic in view of the patients’ clinical phenotype, family history, high predictive metrics of deleteriousness with Polyphen scores of 1 and SIFT scores of 0, and absence from the large population sequencing databases 1000 Genomes (1KG), Exome Sequencing Project (ESP) and Exome Aggregation Consortium (ExAC).

Mutation c.263G > T predicting *UMOD* substitution p.(Gly88Val) occurred in a patient with a diagnosis of medullary cystic kidney disease. A *UMOD* mutation had been found in a relative from another region, although the exact nature of the relative’s mutation was unknown. Mutation c.614 T > C predicting p.(Phe205Ser) was found in a participant with a strong family history of autosomal dominant kidney disease. As the majority of affected relatives lived abroad it was not possible to perform segregation analysis of the variant. Mutation c.860G > A predicting p.(Cys287Tyr) was found in a patient with a diagnosis of FJHN and a strong family history of kidney disease. The same mutation was confirmed in her teenage daughter, who has hyperuricaemia.

### Clinical features

Clinical and biochemical parameters were compared between patients with non-polycystic genetic kidney diseases with and without *UMOD* mutations (Table 4), and between ADTKD patients with and without *UMOD* mutations (Table 5). After Bonferroni correction, patients with ADKTD-UMOD had lower protein creatinine ratios (*p* < 0.001), and a reduced presence of proteinuria (p < 0.001) and haematuria (p < 0.001) compared to all genotyped patients with genetic kidney diseases. There was no statistically significant association between ADTKD-UMOD and age at presentation, age at renal replacement therapy (RRT), gout, allopurinol use, hypertension, hyperuricaemia, uric acid levels, electrolyte abnormalities, anaemia, renal cysts, or renal size. There was a trend for a younger age at presentation for ADTKD-UMOD patients compared to ADTKD-NOS (ADTKD-not otherwise specified, i.e. *UMOD* negative), which lost its statistical significance after Bonferroni correction.

**Table 4:** Comparison of clinical parameters between *UMOD* positive and negative patients with non-polycystic genetic kidney diseases

Clinical parameter	*UMOD* positive	*UMOD* negative	Significance level (*p* < 0.0036)
Age at presentation [years]	9–57, median 39, *n* = 21	0–80, median 35, *n* = 66	*p* = 0.882*
Age at RRT [years]	27–66, median 47, *n* = 27	9–84, median 41, *n* = 61	*p* = 0.116*
Gout	15/33 patients (45%)	30/78 (38%)	*p* = 0.493**
Allopurinol use	13/35 patients (37%)	22/78 (28%)	*p* = 0.342**
Hypertension at presentation	31/35 patients (89%)	69/78 (88%)	*p* = 1.0***
Hyperuricaemia (Uric acid > 0.35 umol/l)	24/26 patients (92%)	50/61 patients (82%)	*p* = 0.328***
Uric Acid [umol/l]	0.28–0.79, median 0.45, *n* = 25	0.12–0.85, median 0.495, *n* = 60	*p* = 0.155*
Proteinuria	8/22 patients (36%)	48/62 patients (77%)	***p*** = 0.0004**
Protein Creatinine Ratio [mg/g]	0–2761, median 234.5, *n* = 18	53–20,398, median = 2150, *n* = 52	p < 0.001*
Anaemia pre RRT (Hb < 100 g/l)	4/27 patients (15%)	25/68 patients (37%)	*p* = 0.036**
Microscopic haematuria	1/27 patients (4%)	24/63 patients (38%)	***p*** = 0.001**
Renal cysts	4/21 patients (19%)	6/51 patients (12%)	*p* = 0.463***
Normal renal size at presentation (renal diameter > 9 cm)	11/23 patients (48%)	30/48 patients (63%)	*p* = 0.241**
Electrolyte abnormalities	6/32 patients (19%)	2/67 patients (3%)	*p* = 0.013***

A Bonferroni correction was employed to adjust the significance level for the number of performed tests (i.e. the adjusted significance level is *p* < 0.05/14)

* = Mann Whitney U, ** = χ^2^, *** = Fisher’s Exact test

**Table 5:** Comparison of clinical parameters between *UMOD* positive and negative patients with ADTKD

Clinical parameter	*UMOD* positive	*UMOD* negative	Significance level (*p* < 0.0036)
Age at presentation [years]	9–57, median 39, *n* = 21	23–80, median 49, *n* = 20	*p* = 0.024*
Age at RRT [years]	27–66, median 47, *n* = 27	27–83, median 51.5, *n* = 16	*p* = 0.606*
Gout	15/33 patients (45%)	10/25 patients (40%)	*p* = 0.678**
Allopurinol use	13/35 patients (37%)	6/25 patients (24%)	*p* = 0.281**
Hypertension at presentation	31/35 patients (89%)	22/25 patients (88%)	p = 1.0***
Hyperuricaemia (Uric acid > 0.35 umol/l)	24/26 patients (92%)	15/19 patients (79%)	*p* = 0.377***
Uric Acid [mg/dl]	4.71–13.28, median 7.75, *n* = 25	4.54–12.27, median 8.07, *n* = 19	*p* = 0.61*
Proteinuria	8/22 patients (36%)	6/17 patients (35%)	*p* = 0.945**
Protein Creatinine Ratio [mg/g]	0–2761, median 234.5, *n* = 18	53–2469, median 624, *n* = 14	*p* = 0.065*
Anaemia pre RRT (Hb < 100 g/l)	4/27 patients (15%)	7/22 patients (32%)	*p* = 0.185***
Microscopic haematuria	1/27 patients (4%)	1/19 patients (5%)	p = 1.0***
Renal cysts	4/21 patients (19%)	4/15 patients (27%)	*p* = 0.694***
Normal renal size at presentation (renal diameter > 9 cm)	11/23 patients (48%)	12/19 patients (63%)	p = 0.32**
Electrolyte abnormalities	6/32 patients (19%)	1/22 patients (5%)	*p* = 0.22***

* = Mann Whitney U, ** = χ^2^, *** = Fisher’s Exact test

### Final prevalence figures

In addition to establishing 13 new diagnoses of ADTKD-UMOD, we identified six additional patients with Alport disease through a targeted next generation sequencing panel of patients with FSGS/SRNS as described previously [36]. In total, we established 35 diagnoses of ADTKD-UMOD in our study population, and 31 diagnoses of Alport disease. 26 patients from 13 families had ADTKD of unknown genotype, and 31 patients were left with an undiagnosed genetic kidney disease.

By counting each family only once, we calculated the population prevalence of ADTKD-UMOD conservatively at 9 per million, and of ADTKD at 16 per million.

## Discussion

This study identified a higher prevalence of genetic kidney diseases than previously described and found ADTKD-UMOD to be the most common genetic kidney disease after ADPKD.

Previous prevalence studies on genetic kidney diseases have largely relied on registry data and have rarely made use of genetic testing. The first study to highlight the importance of familial kidney diseases identified a prevalence of familial glomerulonephritides of 10% of all forms of glomerulonephritis in Germany [37]. An Irish cross-sectional study reported a prevalence of familial kidney diseases other than ADPKD of 4% for transplant and 5% for haemodialysis patients [17]. Similarly, 4% of ESRD patients in Newfoundland [18] and 6% of Swedish transplant patients [20] were reported to have a familial kidney disease other than ADPKD. A single-centre study from Italy established a prevalence of 4% of rare genetic disorders amongst transplant recipients with an unknown diagnosis [21]. A recent registry study of CKD patients of any stage from Australia found a prevalence of genetic kidney diseases other than ADPKD of 6% [38], but controversially included physician-ascertained congenital abnormalities of the kidneys and urinary tract (CAKUT) which constituted two thirds of genetic diagnoses.

Our prevalence of 8% for non-polycystic genetic kidney diseases amongst ESRD patients is higher than the previously published figures. We are the first to give an estimate of genetic kidney diseases amongst patients with CKD3+ of 4% (11% including ADPKD). This figure is lower than in our end-stage population consistent with our finding that patients with genetic kidney diseases were more likely to reach ESRD than patients with other diagnoses, despite being younger.

Our *UMOD* mutation analysis revealed 10 distinct pathogenic mutations in 35 participants from 18 families. Three mutations were unpublished. The presence of affected relatives with a *UMOD* mutation in two of the families makes it highly likely that these are pathogenic mutations, even in the absence of a complete segregation analysis. The third kindred had a strong family history of autosomal dominant kidney disease consistent with ADTKD-UMOD. The maximally deleterious Polyphen and SIFT scores of all three mutations and their absence from the large population databases 1KG, ESP and ExAC lend further support to this interpretation. Mutation c.184A > C predicting p.(Thr62Pro) was considered clinically silent as reported in Ensembl (SNP rs143248111) and in the Wake Forest Registry, and supported by its presence in the non-disease databases ESP and ExAC. Complete segregation analysis in the families was not possible but several affected relatives had variant p.(Thr62Pro) confirmed at another centre.

We are likely to have underestimated the true prevalence of ADTKD-UMOD since we were only able to screen those patients with significant CKD who had been referred to tertiary renal services and their relatives. Conversely, patients with an obvious inherited kidney disease may have been referred to our service earlier than other patients in CKD stage 3. This could theoretically have led to an overestimation of ADTKD as a proportion of CKD patients, but it could not have overestimated the total prevalence figures based on the catchment population. Furthermore, when taking other possible sources of under-ascertainment into account, an overestimation of the prevalence of ADTKD appears very unlikely.

Having only sequenced exons 3–5 in the majority of patients [9, 39], we may have missed mutations in the remaining seven exons of the *UMOD* gene, where only 5% of mutations are expected to occur [40].

The incomplete response rate of 53.8% was a recognised source of incomplete ascertainment. To compensate for this, we undertook a comprehensive database search of all non-responders with missing and/or suspicious diagnostic codes. This search would have captured 79% of responders with ADTKD had they not responded. Furthermore, the prevalence rates established for responders and the cohort as a whole were very similar, indicating that any bias inherent in the different forms of patient ascertainment was likely limited, although a remaining degree of incomplete ascertainment remained.

A limitation of our study is that we have not conclusively established a prevalence for ADTKD since we have only sequenced *UMOD* as the most common underlying mutation [2] and not *MUC1* thought to be responsible for 30% of ADTKD mutations. Furthermore our prevalence rates only apply to a predominantly Caucasian population. Finally, the single centre design is a limitation, although our tertiary renal centre covers a large geographical mixed urban and rural area with a catchment population of 2 million. While a multi-centric design would be preferable, this is the first and only systematic study of the prevalence of ADTKD-UMOD amongst patients with dominant renal disease. No national or international disease registry has been based on a similar systematic approach, explaining the much lower current numbers.

To minimise any distorting local factors such as relatedness of pedigrees, our prevalence rate for ADTKD-UMOD of 9 per million was estimated conservatively by counting each family only once. If we were to count each affected patient instead, we would observe a prevalence of ADTKD-UMOD of 17.5 per million and of ADTKD of 30.5 per million.

We have shown that a simple questionnaire study on family history combined with a database search followed by genetic testing can uncover many additional cases of genetic kidney diseases in general and ADTKD-UMOD in particular.

Apart from incomplete and inadequate coding, the reason for the low published prevalence rates appears to be that genetic kidney diseases often go unrecognized [21]. This is especially true for ADTKD which has subtle phenotypic characteristics that can easily be missed [15]. While genetic tests are available for many genetic kidney diseases, they have not been commonly performed historically, because of their cost and the limited availability of diagnostic centres. Rare disease registries based on genetic results are promising approaches but they still remain in their infancy.

Looking for diagnostic clues, this study has confirmed that clinical and biochemical tests need to be interpreted with caution in the diagnosis of ADTKD-UMOD. Since ADTKD-UMOD is a tubulointerstitial disease, it is not surprising that it was associated less often with haematuria and proteinuria than other genetic kidney diseases, which included familial glomerulonephritides. Despite hyperuricaemia and gout being considered hallmarks of ADTKD-UMOD, there was no significant association with the disease, reflecting how common both are in a general CKD population and that they can be absent in ADTKD-UMOD [9]. Hyperuricaemia and/or gout can still be helpful when present in patients with normal renal function, especially in females and young patients [15].

As we have shown, a positive family history remains the most important diagnostic clue in the diagnosis of ADTKD-UMOD and in genetic kidney diseases in general. However, a family history may be absent in recessive diseases, in de novo mutations and where a relative’s kidney disease was either not diagnosed or communicated to the rest of the family. While we recognise these limitations, we have demonstrated the usefulness of a questionnaire on family history in uncovering many undiagnosed genetic kidney diseases.

In our search for gene mutations, we performed Sanger sequencing of the *UMOD* gene, a targeted next generation sequencing panel of patients with FSGS/SRNS [36], and exome sequencing of selected participants. In future, next generation sequencing techniques such as (clinical) exome and whole genome sequencing are expected to largely replace conventional sequencing. They deliver more genetic information in a single assay and offer superior flexibility, as existing sequencing data can be reviewed once new pathogenic gene mutations become known. However, they bring their own significant problems of the storage and interpretation of large datasets and the interpretation of multiple novel variants. Possible solutions include the segregation analysis of variants of interest, functional studies and pooling of phenotype and genotype data in national and international efforts such as the 100,000 Genomes Project and RaDaR [24, 41]. The 31 study participants currently left with an unknown familial nephropathy will be preferentially recruited to the 100,000 Genomes Project to help uncover their underlying diagnoses which will help to further inform our disease and prevalence data.

## Conclusions

This study has demonstrated that the prevalence of ADTKD, and ADTKD-UMOD in particular, is significantly higher than previously reported. Due to ADTKD’s lack of distinctive clinical features, clinical suspicion should be aroused by a compatible family history alone and should lead to genetic testing. As shown, this approach is able to identify many previously unknown cases of ADTKD-UMOD, which can benefit patients in terms of prognostication, the provision of genetic counselling and the early identification of affected relatives.

## Additional file

Additional file 1: Patient Questionnaire, Word document. (DOCX 18 kb)

## Abbreviations

ADPKD: Autosomal dominant polycystic kidney disease
ADTKD: Autosomal dominant tubulointerstitial kidney disease
ADTKD-UMOD: Autosomal dominant tubulointerstitial kidney disease caused by *UMOD* mutations
CAKUT: Congenital abnormalities of the kidneys and urinary tract
CKD: Chronic kidney disease
ER: Endoplasmic reticulum
ESRD: End stage renal disease
FJHN: Familial juvenile hyperuricaemic nephropathy
FSGS: Focal segmental glomerulosclerosis
HNF1b: Hepatocyte nuclear factor 1 beta
MUC1: Mucin 1
RaDaR: The UK’s National Rare Disease Registry
REN: Renin
RRT: Renal replacement therapy
SRNS: Steroid resistant nephrotic syndrome
UAKD: Uromodulin associated kidney disease
