# Supplementary material for: Autosomal dominant tubulointerstitial kidney disease-UMOD is the most frequent non polycystic genetic kidney disease
Source: BMC Nephrol. 2018 Oct 30;19:301. doi: 10.1186/s12882-018-1107-y (PMC6208030; doi:10.1186/s12882-018-1107-y)
Supplement: Supplementary file 1 — Patient Questionnaire, Word document. (DOCX 18 kb) [file 12882_2018_1107_MOESM1_ESM.docx]

**A STUDY OF FAMILIAL (INHERITED) KIDNEY DISEASES**

Dear Patient,

We are interested in how commonly kidney diseases occur in families and would like to find out more about what is causing kidney diseases to run in families.

We would be very grateful if you could return this slip either by post (by using the stamped addressed envelope), or by handing it in to the receptionists at Renal Outpatients.

- If you know the **diagnosis** of your kidney disease please write it down

……………………………………………………………………………………..

- Are you aware of **any relatives affected by kidney disease** or kidney failure? (This includes someone who may be deceased)

YES (please answer the questions below)

NO (please fill in your details and return the questionnaire as above)

- **If YES, please supply the following information:**

| **Relative (e.g. father/daughter)** | **Kidney disease (where known)** |
| --- | --- |
|  |  |
|  |  |
|  |  |
|  |  |
|  |  |

All the information will be treated in strict confidence. If you have answered YES, we would like to contact you to see if you would be interested in participating in our study.

We may be able to offer the opportunity for special tests (by taking a single blood and/or urine sample) to find out more about the kidney disease affecting your family.

If you have answered YES and are happy to be approached to discuss our study, could you please provide your contact details below? You will find our contact details at the bottom of this page.

Your name.……………………………………………………………………………………..

Address………………………………………………………………………………………...

…………………………………………………………………………………………………..

Email………………………………………… Telephone no………………………………...
